# Action Potential Dynamics During Spreading Depolarization

**DOI:** 10.3390/cells15070602

**Published:** 2026-03-28

**Authors:** Daria Vinokurova, Bulat Mingazov, Gulshat Burkhanova-Zakirova, Roustem Khazipov, Azat Nasretdinov

**Affiliations:** 1 Laboratory of Neurobiology, Kazan Federal University, 420008 Kazan, Russia; daevinokurova@kpfu.ru (D.V.); burmingazov@kpfu.ru (B.M.); gulsfzakirova@kpfu.ru (G.B.-Z.); azrnasretdinov@kpfu.ru (A.N.); 2 Institut de Neurobiologie de la Méditerranée (INMED), L’Institut National de la Santé et de la Recherche Médicale (INSERM), Aix-Marseille University, 13273 Marseille, France

**Keywords:** spreading depolarization, action potential, neuron, cortex, patch-clamp, single unit activity, membrane potential, local field potential, migraine

## Abstract

**Highlights:**

**What are the main findings?**

- During the pre-SD excitation phase, transient spike hyperactivity is accompanied by progressive amplitude reduction and waveform broadening of spikes, culminating in depolarization block.
- These spike dynamic changes are consistently observed across whole-cell, loose cell-attached, and extracellular recordings in cortical neurons.

**What are the implications of the main findings?**

- The characteristic pattern of spike deformation preceding suppression represents a distinct electrophysio-logical feature of SD onset.
- When combined with other SD indicators, this feature may aid in SD detection, particularly in settings where conventional DC recordings are unavailable.

**Abstract:**

Spreading depolarizations (SDs) are major pathophysiological events in several brain diseases, including migraine, brain ischemia, trauma, and epilepsy. However, the electrophysiological detection of SDs remains challenging. In this study, we examined changes in spikes (action potentials (APs) and action currents (ACs)) in layer 5 neurons of the somatosensory cortex of anesthetized rats during transient excitation at the onset of high-potassium-induced SDs. During whole-cell recordings, spike amplitude progressively decreased while spike duration increased during gradual neuronal depolarization at SD onset, culminating in depolarization block. A similar decrease in spike amplitude and increase in spike duration were observed during the pre-SD excitation phase in loose cell-attached recordings from single neurons and in cluster analysis of extracellular spikes. Multiple (non-clustered) unit activity also showed decrease in spike amplitude and spike broadening during pre-SD excitation. These findings suggest that dynamic changes in spike amplitude and duration at SD onset could serve as markers for SD detection.

## 1. Introduction

Spreading depolarization (SD) is a pathophysiological phenomenon characterized by a slowly propagating wave of sustained, nearly complete neuronal depolarization [1,2,3,4,5,6]. It is a key mechanism of migraine aura and a critical determinant of progressive tissue damage in acute brain injuries, including stroke, traumatic brain injury and subarachnoid hemorrhage [7,8,9,10,11,12,13]. The hallmark of SD is a large, negative shift in the local cortical field potential (the “DC shift”), accompanied by a transient suppression of synaptic and neuronal activity, which causes spreading depression of fast electrographic activity [1,2]. These two electrophysiological phenomena, the DC-shift and depression of the ongoing fast EEG and ECoG activity, are the primary diagnostic criteria for SD detection in clinical and experimental studies [14]. However, conventional recordings use high-pass filters that severely suppress DC shifts, whereas depression of fast activity during SD is complicated by variability of changes in fast activity spanning from different levels of depression to even a boost during various SD subtypes [15]. Therefore, SD detection remains challenging, and the search for new reliable SD markers is a major focus of current SD research.

In this context, a brief, few-seconds-long episode of hyperactivity at the SD onset is yet another hallmark of SD. This pre-SD excitation phase, first described by Grafstein, is caused by gradual neuronal depolarization, during which neurons display transient increase in AP firing, followed by arrest in AP firing when the neuronal membrane potential (*E_m_*) reaches values at which AP inactivation occurs [15,16,17,18]. At the network level, the pre-SD excitation phase is organized in low voltage fast (high gamma range) local gamma oscillations in the neocortex and bursts of high-gamma population spikes (also referred to as prodromal spikes) in the hippocampus, supported by non-synaptic mechanisms [15,16,18,19,20,21]. Therefore, pre-SD excitation is a potential SD marker during SD assessments. However, the cellular and network phenomena underlying pre-SD excitation are not fully understood. In particular, since AP features critically depend on the membrane potential dynamics [22], one could expect changes in AP characteristics during gradual neuronal depolarization at the pre-SD excitation phase. In the present study, we aimed at characterizing the dynamic changes in the APs during pre-SD excitation during whole-cell recordings, and ACs (action currents) during cell-attached and spikes during extracellular recordings. Our main finding is that APs, ACs and spikes decrease in amplitude and broaden during gradual depolarization before *E_m_* attains depolarization block. We propose that this characteristic pattern of AP changes could be useful for SD detection when unit recordings are available.

## 2. Materials and Methods

### 2.1. Surgery and Animal Preparation

The animal experiments were conducted in compliance with the ARRIVE guidelines. Animal care and experimental procedures were performed in accordance with EU Directive 2010/63/EU, and all protocols involving animals were approved by the Local Ethical Committee of Kazan Federal University (#24, 22 September 2020).

Wistar rats of both sexes, aged 3 to 5 weeks, were used. Surgical anesthesia was induced and maintained with isoflurane (4% for induction, 2% for maintenance; Aerrane, Baxter, Reading, UK), and the depth of anesthesia was confirmed by the absence of a toe-pinch reflex. The skin overlying the skull and periosteum was removed, and capillary bleeding was controlled with Hemostab (Omegadent, Moscow, Russia), which was subsequently rinsed off with 0.9% NaCl. The incision site was infiltrated with bupivacaine (0.25%). A metal ring was affixed to the skull using dental cement (Meliodent, Heraeus Kulzer, Hanau, Germany). Following this, isoflurane anesthesia was gradually reduced, and animals received an intraperitoneal injection of urethane (Sigma-Aldrich, St. Louis, MO, USA) at a surgical dose (1.5 g/kg), with the maintenance of surgical anesthesia confirmed by immobility and a negative toe-pinch reflex throughout the experiment. Of note, urethane exerts little effect on action potentials in slices in vitro [23] or during anesthesia in vivo [24]. During electrophysiological recordings, rats were placed on a heated platform maintained at 37 °C (TC-344B; Warner Instruments, Hamden, CT, USA). The metal ring was attached via a ball joint to a magnetic stand to restrain head movements. A chloridized silver wire was inserted into the cerebellum or contralateral hemisphere to serve as a ground or reference electrode. A small craniotomy (~0.3 mm in diameter) was drilled above the barrel cortex (approximately 2.5 mm caudal and 5.5 mm lateral to bregma) to allow insertion of a silicon probe. For simultaneous patch-clamp recordings, a second craniotomy (~0.2 mm in diameter) was made within 0.5 mm rostral to the probe insertion site. Another craniotomy (~0.3 mm in diameter) was performed over either the occipital cortex (approximately 6–7 mm caudal and 5–6 mm lateral to bregma) or the frontal cortex (approximately 1–1.5 mm rostral and 3–4.5 mm lateral to bregma) for the induction of spreading depression by application of KCl (0.5–1 M). The dura mater was carefully incised with a sharp needle at the electrode insertion sites. A chamber for KCl application was constructed by building a 1–2 mm high wall of dental cement around the designated craniotomy.

### 2.2. Electrophysiological Recordings and Analysis

Field potential (FP) and multiple unit activity (MUA) were recorded using linear 16-channel or 32-channel (linear, Poly2 (a configuration with two rows of closely spaced electrodes on a single shank)) silicon probes with iridium electrodes (413 μm^2^ surface area; 50 or 100 μm inter-electrode spacing; Neuronexus Technologies, Ann Arbor, MI, USA). The probe was pre-bent at a 20° angle by applying a drop of dental cement at its base and was inserted vertically into the barrel cortex to a depth of 1.6–1.8 mm. Signals were amplified, low-pass filtered at 9 kHz, and digitized at 32 kHz using a DigitalLynx amplifier (Neuralynx, Bozeman, MT, USA). Recordings were performed either (i) in true DC mode (input range ±131 mV) with DC potential offsets compensated at the start of recordings [25], or (ii) using full-band recordings with inverse filtering for signal reconstruction based on hybrid AC/DC-divider RRC filters [26].

Patch-clamp recordings were obtained from layer 5 pyramidal neurons in the barrel cortex at depths of 1000–1300 μm using an Axopatch 200B amplifier (Molecular Devices, San Jose, CA, USA), as previously described [15]. Patch electrodes were pulled from borosilicate glass capillaries (BF150-86-10, Sutter Instrument, Novato, CA, USA) and had resistances of 3.5–5 MΩ for loose cell-attached recordings and 5–7 MΩ for whole-cell recordings. L5 was chosen because it exhibits the highest background neuronal firing level [27,28,29,30]. For whole-cell recordings, pipettes were filled with a solution containing (in mM): 131 potassium gluconate, 4 KCl, 10 HEPES, 10 phosphocreatine, 4 MgATP, and 0.3 Na_2_GTP (pH adjusted to 7.3 with KOH). The patch pipette was inserted into the cortex approximately 0.5 mm from the silicon probe insertion site at an angle of 50–65° directed toward the probe, such that the final distance between the pipette tip and the probe was minimized. Membrane potential values were corrected for a liquid junction potential of 15 mV. Series resistance ranged from 25 to 75 MΩ. Of note, while the series resistance voltage error is minimal under control conditions due to the high membrane resistance, it becomes more pronounced during SD as membrane resistance decreases. Consequently, the observed attenuation of AP amplitude during SD may be overestimated relative to the actual biological change. For loose cell-attached recordings, pipettes were filled with either the same internal solution or artificial cerebrospinal fluid. Patch-clamp recordings were digitized at 40 kHz using a Digidata 1440A interface (Molecular Devices, San Jose, CA, USA).

Raw data were preprocessed using a custom-developed suite of programs in MATLAB 2018b. Positive polarity is plotted upward throughout the manuscript. The original DC signal was downsampled to 1 kHz and used for local field potential (LFP) analysis. SDs with a peak rate of change (SD′) below 1 mV/s were excluded from analysis. SD stop depth was defined as the depth of the deepest electrode exhibiting an SD′ peak greater than 1 mV/s.

In whole-cell recordings, the first derivative of the membrane potential (*E_m_*′) was calculated from the recorded signal and used to detect action potentials’ (APs’) and, consequently, action potentials (APs) as events exceeding a threshold of 20 mV/ms. For each AP, the spike threshold was defined as the time point at which *E_m_*′ crossed 10 mV/ms. The mean membrane potential during the 1 ms preceding the spike threshold was defined as the pre-AP membrane potential. AP amplitude was measured relative to this value, and AP half-duration was determined at half-maximal amplitude. AP’ amplitude and half-duration were calculated from *E_m_*′ as the peak value and the duration at half-maximal amplitude, respectively. The relationships between AP’ amplitude and pre-AP membrane potential, and between AP’ half-duration and pre-AP membrane potential, were fitted with linear and exponential functions, respectively, and the corresponding slope and exponent coefficients were obtained. In whole-cell recordings, membrane potential values were corrected for extracellular voltage shifts during SD by subtracting the LFP recorded from the nearest channel of the silicon probe. In loose cell-attached recordings, AP’ detection was performed on the high-pass filtered (10 Hz) signal as events crossing a threshold of −3 standard deviations. AP’ amplitude and duration were calculated analogously to whole-cell recordings.

Recordings for spike sorting were obtained using high-density silicon probes in either linear or Poly2 configurations. For extracellular spike detection, the original wideband signal was filtered (250–4000 Hz), and negative local peaks exceeding 4 standard deviations of the quietest 100 s fragment during the control period (before the first SD) were considered spikes. Unit detection and clustering were performed automatically using Kilosort4 [31] and manually supervised. For analysis of unit amplitude and half-duration dynamics, values were averaged in 2 s sliding windows with a 100 ms step. For MUA amplitude and half-duration dynamics, averaging was performed in 1 s sliding windows with a 100 ms step.

Numerical data are presented in the text as median and [25th, 75th] percentiles. Statistical analysis was performed using the MATLAB Statistics Toolbox. The Wilcoxon signed-rank test was used to assess the significance of differences between samples. Correlation levels were calculated as the Pearson correlation coefficient with exact *p*-values. The significance level was set at *p* < 0.05.

## 3. Results

### 3.1. Whole-Cell Recordings

**Figure 1:**
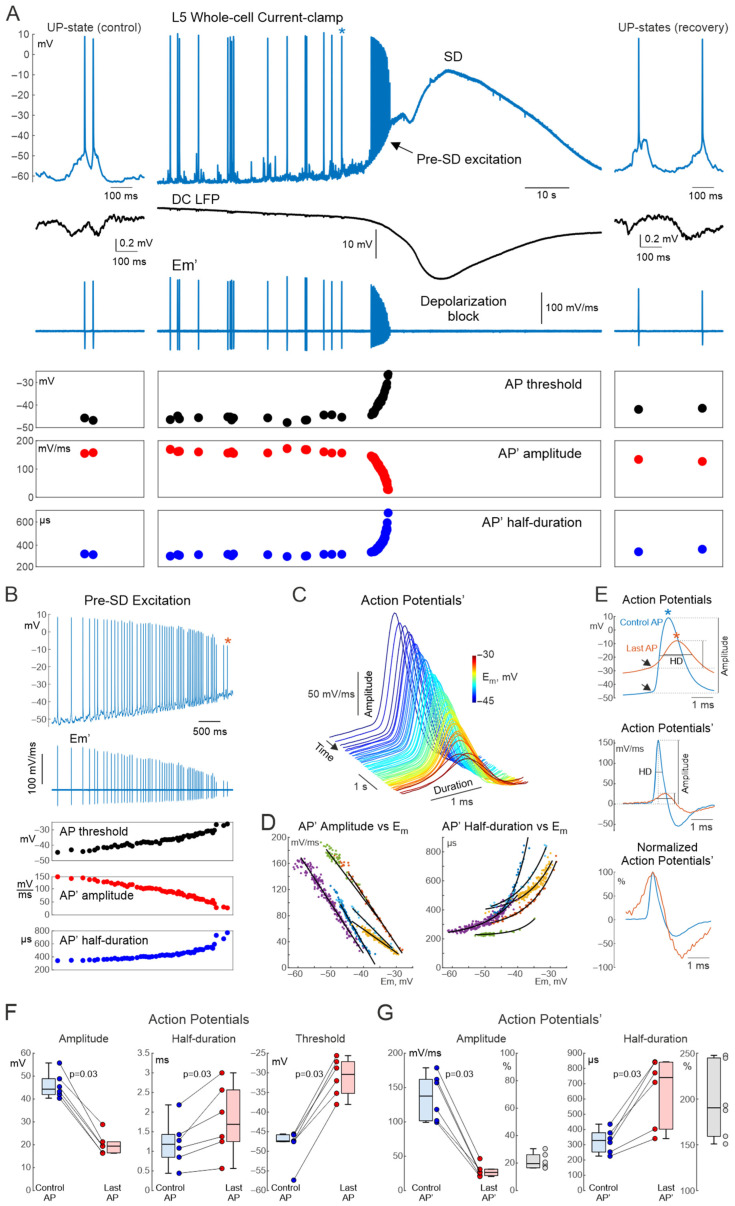
Whole-cell recordings of rat cortical neurons during spreading depolarization. (**A**) From top to bottom: representative recording from a rat layer 5 cortical neuron in whole-cell current-clamp mode during potassium-induced spreading depolarization (*E_m_*, blue trace, action potentials (AP)); simultaneous local field potential recording (black trace); corresponding fragment of the first derivative of *E_m_* (*E_m_*′) and action potentials’ (AP’); spike threshold values (black dots); corresponding AP’ amplitude and half-duration values (red and blue dots). Left panels show an example up-state during the control period before SD onset; right panels show an example up-state during recovery after SD. The blue asterisk in the top panel indicates the control AP selected as an example in panel (**E**). (**B**) Pre-SD excitation phase shown at an expanded time scale for the same set of parameters as in panel (**A**). The red asterisk in the top panel indicates the last AP selected as an example in panel (**E**). (**C**) 3D representation of AP’ during pre-SD excitation, demonstrating progressive reduction in AP’ amplitude and increase in AP’ duration over time as depolarization develops during SD. Axes: x—AP’ duration (ms), y—AP’ timestamp (s), z—AP’ amplitude (mV/ms). Color code represents the mean pre-AP membrane potential during the 1 ms preceding spike threshold. (**D**) Dependence of AP’ amplitude (left) and half-duration (right) on pre-AP membrane potential for six cells, each cell has its own color on the graph Linear fits are shown for amplitude, exponential fits for half-duration. (**E**) Top panel: representative control and last AP illustrating the measurements of AP amplitude and half-duration; arrows indicate spike thresholds. Middle panel: corresponding AP’ waveforms with indicated amplitudes and half-durations. Bottom panel: control and last AP’ normalized to the maximal value. (**F**) Boxplots showing changes in amplitude, half-duration, and spike threshold of the last AP relative to control values. (**G**) Boxplots showing changes in amplitude and half-duration of the last AP’ compared to control; gray indicates normalized values (Last AP’/Control AP’). Group data: *n* = 6 cells from 6 animals.

To characterize AP dynamics during SD, whole-cell recordings from L5 neurons concomitant with extracellular DC-LFP recordings were performed from the rat somatosensory cortex during SDs evoked by distal high-potassium application. As illustrated in the representative recordings, under control conditions, prior to SD, neuronal *E_m_* fluctuated between the up- and down-states in association with delta-wave oscillations, and occasionally fired APs during the up-states (Figure 1A, left panel). Consistent with previous findings [15,16,17,18], the SD onset was associated with a transient elevation of AP frequency along with a gradual *E_m_* depolarization followed by an abrupt arrest in APs and total AP suppression along with nearly complete loss of the *E_m_* during SD (Figure 1A). After SD, APs recovered after a delay after neuronal repolarization (Figure 1A, right panel). During the pre-SD excitation phase, APs displayed a progressive decrease in their amplitude and an increase in duration along with gradual depolarization at the SD onset (Figure 1A,B). On average, the AP amplitude decreased from 44.3 [42.0, 48.8] mV to 19.4 [16.5, 21.2] for the last AP during the pre-SD excitation phase (*p* = 0.03, *n* = 6 cells from 6 animals, Figure 1F). The AP half-duration, in turn, increased from 1.18 [0.85, 1.42] to 1.69 [1.25, 2.56] ms (*p* = 0.03, Figure 1F). Also, the AP threshold shifted towards depolarizing *E_m_* values from −47.37 [−47.53, −45.81] to −30.40 [−35.20, −27.16] mV (*p* = 0.03, Figure 1F). To translate these findings to extracellular spikes, the first derivative of APs (AP’), whose waveform is similar to extracellular spikes [32,33] was calculated. The AP’ amplitude and half-duration showed similar changes during the phase of gradual depolarization at the SD onset (Figure 1A–E). The AP’ amplitude reduced from 137.6 [101.8, 161.8] mV/ms to 26.4 [21.9, 31.0] mV/ms (*p* = 0.03), and the AP’ half-duration increased from 328 [253, 378] µs to 741 [403, 841] µs (Figure 1G). Whereas the dependence of the AP’ amplitude on *E_m_* during SD was nearly linear with a slope of −7.1 [−7.7, −5.0] (mV/ms)/mV (Figure 1D, left panel), the AP’ widening with depolarization was characterized by steeper dependence of AP’ half-duration on *E_m_* at more depolarized *E_m_* values, and it was better matched by an exponential fit with an exponent of 0.123 [0.122, 0.190] mV^−1^ (Figure 1D, right panel). Thus, whole cell recordings revealed remarkable changes in AP and AP’ amplitude and waveform during gradual depolarization at the SD onset. We further verified whether these changes also appear during extracellular recordings.

### 3.2. Loose-Cell Attached Recordings

**Figure 2:**
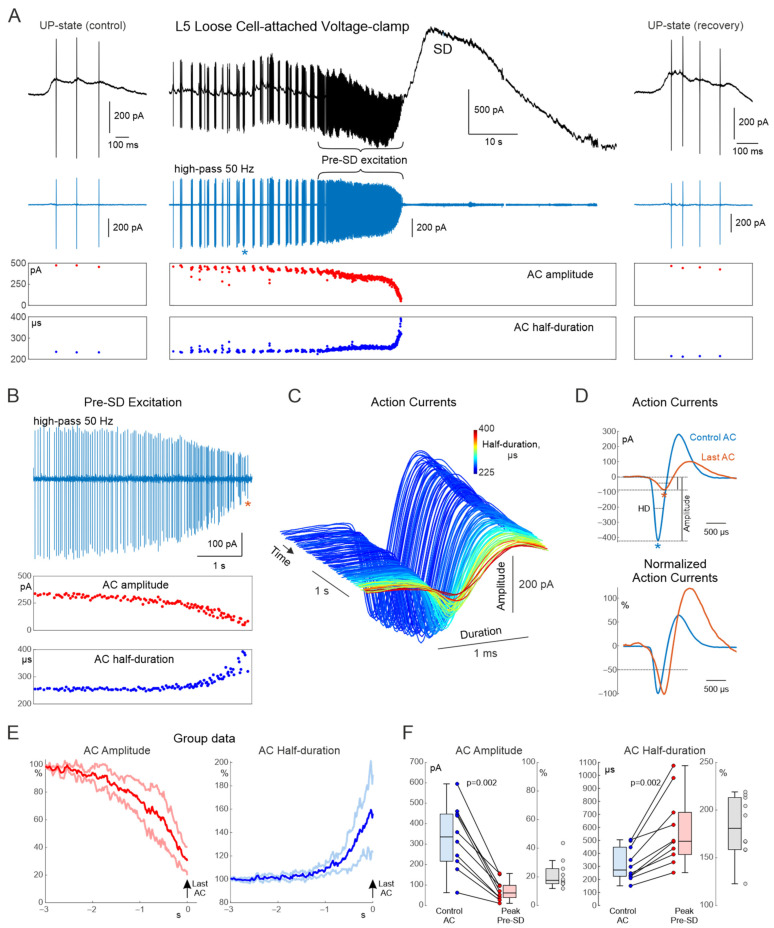
Loose cell-attached recordings of rat cortical neurons during spreading depolarization. (**A**) From top to bottom: representative recording from a rat layer 5 cortical neuron in loose cell-attached voltage-clamp configuration during potassium-induced spreading depolarization (black trace); corresponding high-pass filtered trace (>50 Hz; blue trace). The blue asterisk indicates the control AC selected as an example in panel (**D**). Red and blue dots represent corresponding AC amplitude and half-duration values. Left panels show an example up-state during the control period before SD onset; right panels show an example up-state during recovery after SD. (**B**) Pre-SD excitation phase shown at expanded time scale from panel (**A**). The red asterisk in the top panel indicates the AP selected as an example in panel (**D**). (**C**) 3D representation of action currents during pre-SD excitation, demonstrating progressive reduction in AC amplitude and increase in AC duration during depolarization. Axes: x—AC duration (ms), y—AC timestamp (s), z—AC amplitude (pA). Color code represents the half-duration of the corresponding AP. (**D**) Top panel: representative control and pre-SD (last) AC illustrating measurements of amplitude and AP half-duration (HD);. Bottom panel: control and pre-SD AC normalized to the negative peak value. (**E**) Mean (central trace) ± sd (light color) of AC amplitude and half-duration normalized to control (group data: *n* = 10 cells from 4 animals), plotted relative to t = 0 corresponding to the last AC within a pre-SD excitation burst. (**F**) Boxplots showing peak amplitude and half-duration values of pre-SD AC compared to control values; gray indicates normalized values (pre-SD AC/Control AC). Group data: *n* = 10 cells from 4 animals.

We next assessed AC changes at the SD onset during loose cell-attached, voltage-clamp recordings that enable reliable recording of ACs from a single neuron with high signal/noise ratio. In addition, LFP signals also contribute to the loose-cell attached recordings as shown in the representative recordings from a L5 barrel cortex neuron (Figure 2A). Of note, unlike inwardly directed capacitance ACs, extracellular LFP signals in this recording configuration are inverted in polarity, with the up-states (Figure 2A, left panel) and SD (Figure 2A, central panel) showing positive polarity as during extracellular recordings in voltage-clamp mode [34]. Consistent with the results obtained using whole-cell recordings, the frequency of ACs during loose cell-attached recordings transiently increased at the SD onset followed by complete suppression of neuronal firing during SD. Throughout the pre-SD excitation phase, the AC amplitude progressively decreased while the AC duration increased (Figure 2A–D). Because *E_m_* values are not available in loose cell-attached recordings, the AC parameters were aligned in time taking the last pre-SD AC as a time = 0 reference point, revealing the characteristic gradual decrease in AC amplitude and increase in AC duration over the pre-SD excitation phase (Figure 2E). At the group level, the AC amplitude reduced from the control values of 335 [216, 447] to 61 [39, 98] pA (*p* = 0.002; *n* = 10 cells from 4 animals), and the AC half-duration increased from 272 [225, 447] to 494 [392, 715] µs for the last AC in the pre-SD burst (*p* = 0.002; Figure 2F).

### 3.3. Extracellular Recordings of Single Units

**Figure 3:**
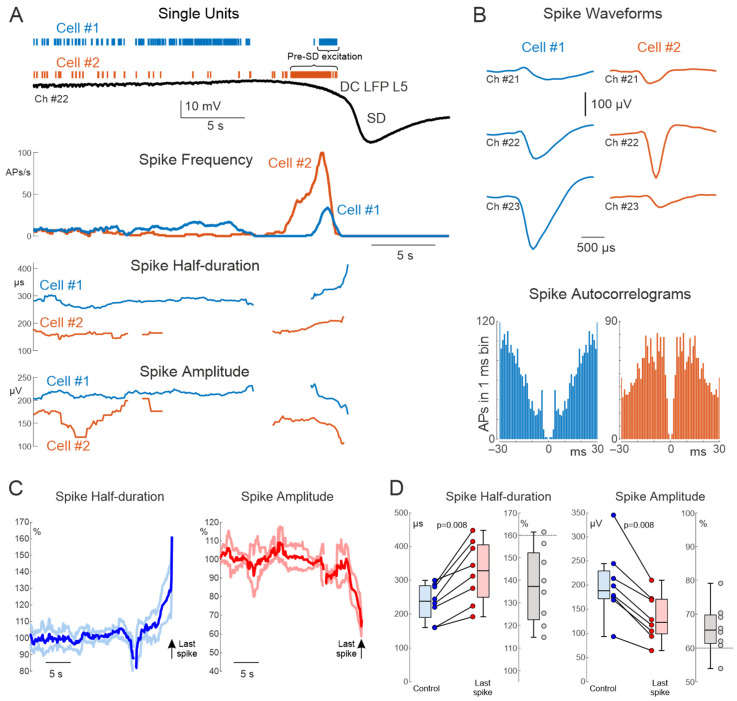
Single-unit recordings using high-density silicon probes in the rat cortex during spreading depolarization. (**A**) Representative DC LFP recording in rat layer 5 barrel cortex from one channel of a silicon probe. Vertical ticks indicate spike times of a putative pyramidal neuron (blue) and interneuron (red). Below are the corresponding temporal dynamics of firing rate, half-duration, and spike amplitude. (**B**) Average unit waveforms simultaneously recorded on three channels of the silicon probe and corresponding autocorrelograms. (**C**) Mean (central trace) ± sd (light color) of half-duration and amplitude normalized to control (group data: *n* = 8 cells from 6 animals), plotted relative to t = 0 corresponding to the last unit within a pre-SD excitation burst. (**D**) Boxplots showing peak half-duration and amplitude values of units during the pre-SD excitation phase compared to control; gray indicates normalized values (pre-SD/Control). Group data: *n* = 8 cells from 6 animals. Horizontal dashed lines indicate cluster loss levels obtained by simulation with predefined changes in unit waveform shape and spike timing.

Extracellular recordings using high-density, high impedance electrodes enable identification of spikes from individual neurons during multiple unit recordings through the cluster analysis. Example spikes from two single units, one putative L5 pyramidal cell and one putative fast-spiking interneuron recorded at three neighboring electrodes of a silicon probe are shown in Figure 3A. Single units were clustered automatically using Kilosort4 [31] (Figure 3B). All neurons showed a transient increase in firing with a characteristic gradual decrease in spike amplitude and increase in spike duration at the SD onset (Figure 3A) like the results obtained during whole-cell and loose cell-attached recordings (Figure 3C). Due to the small number of detected units and the relatively low signal-to-noise ratio, averaged values from 2 s windows were analyzed. As a result, the level of maximal spike amplitude depression (from 188 [172, 229] to 123 [100, 170] µV, *p* = 0.008; *n* = 8 cells from 6 animals) was lower than during whole-cell and loose cell-attached recordings (65% of control values for single units clustered from extracellular recordings vs. 19% and 18% during whole-cell and cell-attached recordings, respectively; Figure 3D). The level of spike half-duration increase was also lower (from 238 [190, 284] to 328 [250, 405] µs), i.e., 38% whereas during whole-cell and loose cell-attached recordings this level was 126% and 81% respectively; Figure 3D). This difference may also be due to changes in the amplitude and shape of units during depolarization, which may lead the spike-sorting algorithm to exclude modified units from the cluster. A similar problem has also been described in studies on AP changes during epileptic discharges, where the spikes escape from clusters because of a reduction in amplitude and broadening [35,36,37]. To identify the threshold levels for cluster loss due to changes in unit shape, a simulation was conducted in which the amplitude decreased and the half-duration of units increased according to the dependences in Figure 1D. It was found that, as depolarization progressed, the last detected unit had an amplitude of 60% and a half-duration of 160% of the control values (horizontal dashed lines in Figure 3D), while subsequent spikes were assigned to a different cluster or interpreted as noise events.

### 3.4. Extracellular Recordings of Multiple Units

**Figure 4:**
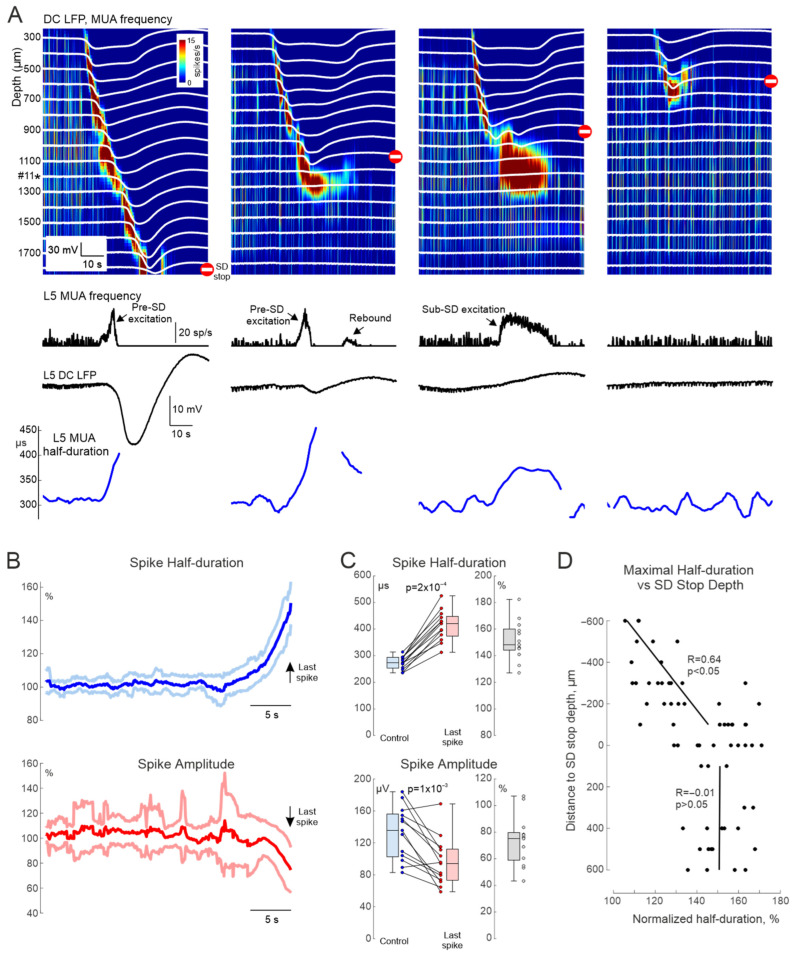
Multiple-unit activity recordings using silicon probes in the rat cortex during spreading depolarization. (**A**) Representative DC LFP and MUA frequency recordings in rat barrel cortex from 16 channels of a multichannel silicon probe. Four examples with different SD stop depths are shown: (1) SD propagating through all cortical layers; (2) SD stopped in layer 5; (3) SD stopped in layer 4; (4) SD stopped in layer 2/3. Traces in the lower panels show MUA frequency, DC LFP, and MUA half-duration dynamics from the selected channel in layer 5 (the asterisk indicates the channel (#11, depth 1200 µm) for which MUA half-duration was calculated). (**B**) Mean (central trace) ± sd (light color) of MUA half-duration and amplitude normalized to control (group data: *n* = 13 SDs from 13 animals), plotted relative to t = 0 corresponding to the last MUA within a pre-SD excitation burst. (**C**) Boxplots showing peak half-duration and amplitude values of MUA during the pre-SD excitation phase compared to the control; gray indicates normalized values (pre-SD/Control). Group data: *n* = 13 SDs from 13 animals. (**D**) Plot of relative change in MUA half-duration in layer 5 as a function of distance to SD stop depth. Negative y-axis values (−600 to −100 µm) correspond to SD stopped above the recording channel (superficial partial SDs; examples 2–3 in panel (**A**)). Positive values (100–600 µm) correspond to SD propagating deeper than the recording channel (deep partial SD and full SD; example 1 in panel (**A**)). Each dot corresponds to the depth of the SD stop relative to the selected layer 5 channel (repeated depths were averaged in each of the animals). Linear fits and Pearson correlation coefficients are shown for each group.

Finally, we explored changes in spikes from extracellular multiple unit activity (MUA) recordings. Consistent with previous studies and the results above, MUA showed a transient elevation in frequency during the pre-SD excitation phase followed by abrupt MUA cessation during SD (Figure 4A). While MUA consistently showed an increase in spike duration during the pre-SD phase, changes in spike amplitude were variable (Figure 4B,C). On average, spike half-duration of MUA in the last 1 s window of the pre-SD burst was 54% longer than that of the control values (273 [253, 293] µs vs. 420 [373, 447] µs, *p* = 0.0002; *n* = 13 SDs from 13 animals), and the amplitude was 70% of the control values (136 [103, 156] µV vs. 94 [73, 112] µV, *p* = 0.001; (Figure 4C)). Despite the tendency for the MUA amplitude to decrease at the end of the SD pre-excitation, it demonstrated higher variability even before the onset of the SD (Figure 4B). Nevertheless, both the prolongation of MUA duration and decrease in its amplitude during pre-SD excitation may also serve as markers of SD.

### 3.5. Full and Partial SDs

SDs vary in their propagation from the surface to the cortical depth and include phenotypes of full SDs invading all cortical layers and partial SDs preferentially invading superficial cortical layers [15,25,38,39,40,41,42,43,44]. While full SDs are characterized by pre-SD excitation at all depths, partial SDs are associated with dynamic cortical states including sub-SD excitation just below the SD stop depth, where neurons display moderate depolarization and sustained firing during the SD above. To characterize the changes in spikes during SDs of different SD phenotypes, MUA were analyzed in L5 with reference to the SD stop depth relative to the recording site. Representative recordings of four SDs stopping at different depths and corresponding MUA frequency and MUA half-duration are shown in Figure 4A. In cases of full SD and partial SD stopping 300–500 µm below L5, pre-SD excitation was associated with an increase in the MUA half-duration, followed by suppression of spikes during SD in L5. Of note, while full SD was associated with a long-lasting depression of spikes, during SD stopping 100 µm above L5 spikes were suppressed for a shorter time followed by rebound spikes that were wider than control spikes. Partial SD that stopped 200–300 µm above L5 was associated with sustained L5 excitation (“sub-SD excitation” [15,39]) and increase in spike half-duration. Finally, in a case of partial SD stopping 500–600 µm above L5, neither spike frequency nor their half-duration changed during SD above. Group data analysis revealed that the peak values of the L5 spike half-duration during SD stopping above L5 correlated with the vertical distance between the recording site in L5 and SD stop depth (*R* = 0.68; *p* < 0.05; Figure 4D). For the cases of SD invading L5, the half-duration of L5 spikes at the end of pre-SD excitation, that is before inactivation, attained maximal values and did not depend on the further SD penetration depth (R = −0.01; *p* > 0.05; Figure 4D). These results are consistent with variety in depolarization levels in L5 neurons during various types of SDs depending on vertical distance from the neuron to the SD stop depth [15].

## 4. Discussion

The main finding of the present study is that APs and ACs gradually decrease in amplitude and increase in duration during pre-SD excitation at the SD onset, before the transition to depolarization block, and these can serve as an additional marker of SDs.

During whole-cell recordings, we found that gradual neuronal depolarization at the SD onset is associated with a transient increase in neuronal firing, as well as a reduction in AP amplitude and an increase in AP duration during the pre-SD excitation phase. This culminates in an abrupt cessation of neuronal firing when *E_m_* reaches values associated with depolarization block. Changes in AP amplitude and dynamics, as well as the depolarizing shift in AP threshold at SD onset, are consistent with the *E_m_*—dependent changes in the APs during prolonged depolarization and repetitive firing [45,46]. These changes involve inactivation of voltage-gated sodium channels [47,48,49,50,51], and potassium channels [52,53,54]. During SD, additional factors may involve disturbances in the ion gradients, notably potassium and sodium, resulting in a decrease in the driving forces for sodium and potassium [6,55]. A similar reduction in the amplitude, and broadening, of ACs at SD onset was also observed during loose cell-attached and extracellular recordings of single units. ACs broadening was also prominent during extracellular recordings of multiple units, although the reduction in their amplitude did not look entirely reliable. The differences in the amplitude decrease and half-duration increase observed in whole-cell and loose cell-attached recordings versus single-unit and MUA recordings are most likely primarily attributable to the lower signal-to-noise ratio inherent to extracellular recordings. This also necessitates averaging in sliding windows, which may lead to deviations from the true values of the last spike in a burst. In addition, spike-sorting algorithms typically rely on classification of units based on waveform shape and amplitude–temporal features. During SD, these parameters undergo substantial changes, which inevitably lead to progressive loss of units from clusters as depolarization develops.

The changes in APs and extracellular spikes described here at the SD onset could serve as one of the markers of SD during their detection in clinical settings. As explicated in the Introduction, the DC shifts in extracellular voltage—considered the gold standard in SD detection—require DC recordings, which are not always available. Another robust SD marker—depression of activity (mainly in the delta-frequency range)—is also not a ubiquitous and specific SD marker [15]. In this context, pre-SD excitation—characterized by a transient increase in neuronal firing, a progressive decrease in spike amplitude, and spike broadening—precedes the SD-related DC shift. This pattern could be considered a potential SD marker during intracortical recordings in patients where DC recordings are unavailable but unit recordings are feasible. This approach for SD detection, based on the characteristic pre-SD excitation phenotype curtailed by long-lasting spike suppression during SD, could also be indicative of SD in purely spike recordings. Since pre-SD excitation is likely a pathophysiological substrate of the positive symptoms of aura (with or without migraine) followed by an SD-evoked depression of activity as a substrate of the negative symptoms of aura [3,56,57,58], it would be of interest to test whether these aura symptoms are associated with a slowly propagating wave of transient bursts of spikes, with a characteristic decline in amplitude and broadening prior to cessation, in cortical regions responsible for corresponding aura hallucinations. This approach could also be adopted in other pathologies associated with SD, including brain trauma [59,60], subarachnoid hemorrhage [12], focal and global ischemia (including terminal ischemia) [7,61], brain cancer [62], encephalitis [63], electrostimulation [1,64], and epilepsy [63]. Such investigations would require spike recordings using implanted microelectrodes, such as silicon-based microelectrode Utah arrays, or microwire bundles like the Behnke–Fried array, which are typically used in patients undergoing epilepsy monitoring or deep brain stimulation surgery [35,37,63,65]. This may also include a population of tetraplegic patients treated with Brain–Machine Interface (BMI) applications, where single-unit recordings are predominantly achieved using the Utah array [66,67]. Beyond the miniature size of electrodes required for spike detection, spike recordings also demand high frequency bandpass (~10 kHz) and sampling rates (~30 kHz). Large electrodes for intracortical recordings, as well as ECoG or EEG electrodes, are poorly suited for spike recordings [33]. In future studies, it will be important to verify whether the pattern of spike changes at SD onset is specific to SD, as similar changes in spikes—including progressive amplitude decrease and broadening eventually followed by depolarization block—are expected during any condition involving strong neuronal depolarization, such as paroxysmal depolarizing shifts [35,36,37]. In this regard, the relatively slow velocity of SD propagation (5–10 mm/min) could be instructive for discriminating the underlying pathophysiological event.

## 5. Conclusions

Thus, the pre-SD excitation phase is characterized by a stereotyped sequence of spike dynamics—progressive amplitude reduction, waveform broadening, and ultimate depolarization block—that is robustly observed across whole-cell, loose cell-attached, and extracellular recording configurations in cortical neurons. Importantly, the consistent temporal pattern of transient spike hyperactivity associated with gradual spike deformation and followed by spike suppression provides a reliable electrophysiological signature that can serve as a practical marker for SD detection. The ability to identify SDs through spike dynamics may help bridge cellular events with their neurological manifestations, offering a valuable tool for SD detection in both basic research and clinical monitoring, particularly in settings where conventional DC recordings are unavailable, in conditions such as migraine, trauma, and ischemia.

## Data Availability

Data supporting reported results are available from the authors upon reasonable request.

## Abbreviations

The following abbreviations are used in this manuscript:

SD	Spreading Depolarization
AP	Action potential
AC	Action current
DC	Direct current
EEG	Electroencephalography
ECoG	Electrocorticography
*E_m_*	Membrane potential
MUA	Multiple unit activity
